# 2-Methyl-3-nitro­benzonitrile

**DOI:** 10.1107/S1600536808039354

**Published:** 2008-11-29

**Authors:** Guo-zhi Han, Lu-na Han, Ran-zhe Lu, Min Zhang, Hai-bo Wang

**Affiliations:** a College of Science, Nanjing University of Technology, Xinmofan Road No. 5 Nanjing, Nanjing 210009, People’s Republic of China

## Abstract

The asymmetric unit of the title compound, C_8_H_6_N_2_O_2_, contains two independent mol­ecules, the aromatic rings of which are oriented at a dihedral angle of 1.68 (3)°. Intra­molecular C—H⋯O hydrogen bonds result in the formation of two non-planar six-membered rings, which adopt envelope and twisted conformations. In the crystal structure, inter­molecular C—H⋯O hydrogen bonds link the mol­ecules. There are π–π contacts between the benzene rings [centroid–centroid distances = 3.752 (3) and 3.874 (3) Å].

## Related literature

For general background, see: Suzuki *et al.* (1994 ▶). For a related structure, see: Xinhua *et al.* (2003 ▶). For bond-length data, see: Allen *et al.* (1987 ▶).
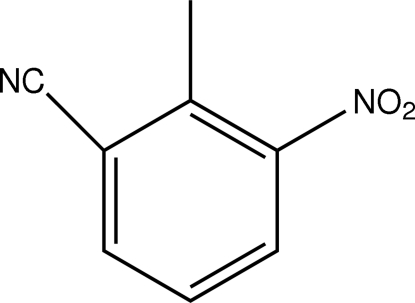


## Experimental

### 

#### Crystal data

C_8_H_6_N_2_O_2_
*M* *_r_* = 162.15
Monoclinic, 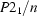

*a* = 14.025 (3) Å
*b* = 7.3860 (15) Å
*c* = 15.515 (3) Å
β = 101.80 (3)°
*V* = 1573.2 (6) Å^3^
*Z* = 8
Mo *K*α radiation
μ = 0.10 mm^−1^
*T* = 294 (2) K
0.30 × 0.20 × 0.10 mm

#### Data collection

Enraf–Nonius CAD-4 diffractometer
Absorption correction: ψ scan (North *et al.*, 1968 ▶) *T*_min_ = 0.970, *T*_max_ = 0.990
2974 measured reflections
2852 independent reflections
1481 reflections with *I* > 2σ(*I*)
*R*_int_ = 0.069
3 standard reflections frequency: 120 min intensity decay: 1%

#### Refinement

*R*[*F* ^2^ > 2σ(*F* ^2^)] = 0.076
*wR*(*F* ^2^) = 0.170
*S* = 1.01
2852 reflections
217 parameters
H-atom parameters constrained
Δρ_max_ = 0.25 e Å^−3^
Δρ_min_ = −0.27 e Å^−3^

### 

Data collection: *CAD-4 Software* (Enraf–Nonius, 1989 ▶); cell refinement: *CAD-4 Software*; data reduction: *XCAD4* (Harms & Wocadlo, 1995 ▶); program(s) used to solve structure: *SHELXS97* (Sheldrick, 2008 ▶); program(s) used to refine structure: *SHELXL97* (Sheldrick, 2008 ▶); molecular graphics: *SHELXTL* (Sheldrick, 2008 ▶); software used to prepare material for publication: *SHELXTL*.

## Supplementary Material

Crystal structure: contains datablocks global, I. DOI: 10.1107/S1600536808039354/hk2576sup1.cif

Structure factors: contains datablocks I. DOI: 10.1107/S1600536808039354/hk2576Isup2.hkl

Additional supplementary materials:  crystallographic information; 3D view; checkCIF report

## Figures and Tables

**Table 1:** Hydrogen-bond geometry (Å, °)

*D*—H⋯*A*	*D*—H	H⋯*A*	*D*⋯*A*	*D*—H⋯*A*
C1—H1*A*⋯O2	0.96	2.08	2.768 (6)	128
C1—H1*C*⋯O2^i^	0.96	2.38	3.229 (6)	147
C4—H4*A*⋯O1^ii^	0.93	2.47	3.390 (6)	171
C9—H9*B*⋯O4	0.96	2.35	2.831 (5)	110
C9—H9*C*⋯O3^iii^	0.96	2.50	3.400 (4)	156

Symmetry codes: (i) 
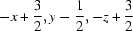
; (ii) 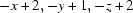; (iii) 
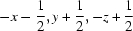
.

**Fig. 1.**
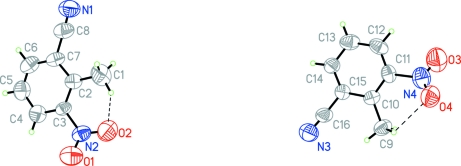
The molecular structure of the title molecule, with the atom-numbering scheme. Displacement ellipsoids are drawn at the 30% probability level. Hydrogen bonds are shown as dashed lines.

## supplementary crystallographic information

### Comment

Benzonitrile is an important pharmaceutical intermediate, many of its derivatives have biological activity and be used as a variety of drugs. Benzonitrile was found to be almost inert toward the combined action of nitrogen dioxide and dioxygen at room temperature (Suzuki *et al.*, 1994). We report herein the crystal structure of the title compound.

The asymmetric unit of the title compound, (Fig. 1), contains two crystallographically independent molecules, in which the bond lengths (Allen *et al.*,  1987) and angles are within normal ranges. Rings A (C2-C7) and B (C10-C15) are, of course, planar, and they are oriented at a dihedral angle of 1.68 (3)°. The intramolecular C-H···O hydrogen bonds result in the formation of two nonplanar six-membered rings C (O2/N2/C1-C3/H1A) and D (O4/N4/C9-C11/H9B). Ring C adopts envelope conformation with O2 atom displaced by 0.690 (3) Å from the plane of the other ring atoms, while ring D has twisted conformation.

In the crystal structure, intermolecular C-H···O hydrogen bonds (Table 1) link the molecules, in which they may be effective in the stabilization of the structure. The π-π contacts between the benzene rings Cg1···Cg1^i^ and Cg1···Cg2^ii^ [symmetry codes: (i) -x, 2 - y, -z, (ii) 1/2 - x, 1/2 + y, 1/2 - z, where Cg1 and Cg2 are centroids of the rings A (C2-C7) and B (C10-C15), respectively] may further stabilize the structure, with centroid-centroid distances of 3.752 (3) %A and 3.874 (3) %A, respectively.

### Experimental

The title compound is synthesized according to the literature method (Xinhua *et al.*,  2003). Crystals suitable for X-ray analysis were obtained by slow evaporation of an ethanol solution.

### Refinement

H atoms were positioned geometrically, with C-H = 0.93 and 0.96 Å for aromatic and methyl H and constrained to ride on their parent atoms, with U_iso_(H) = xU_eq_(C), where x = 1.2 for aromatic H and x = 1.5 for methyl H atoms.

### Crystal data

**Table:**

C_8_H_6_N_2_O_2_	*F*_000_ = 672
*M**_r_* = 162.15	*D*_x_ = 1.369 Mg m^−^^3^
Monoclinic, *P*2_1_/*n*	Mo *K*α radiation λ = 0.71073 Å
Hall symbol: -P 2yn	Cell parameters from 25 reflections
*a* = 14.025 (3) Å	θ = 9–12º
*b* = 7.3860 (15) Å	µ = 0.10 mm^−^^1^
*c* = 15.515 (3) Å	*T* = 294 (2) K
β = 101.80 (3)º	Block, colorless
*V* = 1573.2 (6) Å^3^	0.30 × 0.20 × 0.10 mm
*Z* = 8	

### Data collection

**Table:**

Enraf–Nonius CAD-4 diffractometer	*R*_int_ = 0.069
Radiation source: fine-focus sealed tube	θ_max_ = 25.3º
Monochromator: graphite	θ_min_ = 1.8º
*T* = 294(2) K	*h* = 0→16
ω/2θ scans	*k* = 0→8
Absorption correction: ψ scan(North *et al.*, 1968)	*l* = −18→18
*T*_min_ = 0.970, *T*_max_ = 0.990	3 standard reflections
2974 measured reflections	every 120 min
2852 independent reflections	intensity decay: 1%
1481 reflections with *I* > 2σ(*I*)	

### Refinement

**Table:**

Refinement on *F*^2^	Secondary atom site location: difference Fourier map
Least-squares matrix: full	Hydrogen site location: inferred from neighbouring sites
*R*[*F*^2^ > 2σ(*F*^2^)] = 0.076	H-atom parameters constrained
*wR*(*F*^2^) = 0.170	*w* = 1/[σ^2^(*F*_o_^2^) + (0.04*P*)^2^ + 1.75*P*] where *P* = (*F*_o_^2^ + 2*F*_c_^2^)/3
*S* = 1.01	(Δ/σ)_max_ < 0.001
2852 reflections	Δρ_max_ = 0.25 e Å^−^^3^
217 parameters	Δρ_min_ = −0.27 e Å^−^^3^
Primary atom site location: structure-invariant direct methods	Extinction correction: none

### Special details

**Table:**

Geometry. All esds (except the esd in the dihedral angle between two l.s. planes) are estimated using the full covariance matrix. The cell esds are taken into account individually in the estimation of esds in distances, angles and torsion angles; correlations between esds in cell parameters are only used when they are defined by crystal symmetry. An approximate (isotropic) treatment of cell esds is used for estimating esds involving l.s. planes.
Refinement. Refinement of F^2^ against ALL reflections. The weighted R-factor wR and goodness of fit S are based on F^2^, conventional R-factors R are based on F, with F set to zero for negative F^2^. The threshold expression of F^2^ > 2sigma(F^2^) is used only for calculating R-factors(gt) etc. and is not relevant to the choice of reflections for refinement. R-factors based on F^2^ are statistically about twice as large as those based on F, and R- factors based on ALL data will be even larger.

### Fractional atomic coordinates and isotropic or equivalent isotropic displacement parameters (Å^2^)

**Table:**

	*x*	*y*	*z*	*U*_iso_*/*U*_eq_	
O1	0.8903 (3)	0.3923 (5)	0.9651 (2)	0.1022 (11)	
O2	0.8075 (3)	0.2944 (5)	0.8476 (2)	0.1053 (12)	
O3	−0.2355 (3)	−0.1305 (6)	0.2733 (2)	0.1165 (13)	
O4	−0.2358 (2)	0.0205 (5)	0.3874 (2)	0.0933 (10)	
N1	0.9792 (3)	−0.4285 (6)	0.7823 (3)	0.0911 (13)	
N2	0.8813 (3)	0.2924 (5)	0.9055 (2)	0.0679 (9)	
N3	0.1646 (3)	0.3683 (6)	0.4808 (3)	0.0913 (12)	
N4	−0.1954 (3)	−0.0405 (6)	0.3328 (3)	0.0848 (12)	
C1	0.8268 (3)	−0.0774 (7)	0.8368 (3)	0.0923 (16)	
H1A	0.7835	0.0171	0.8470	0.139*	
H1B	0.8181	−0.1816	0.8713	0.139*	
H1C	0.8128	−0.1092	0.7755	0.139*	
C2	0.9291 (3)	−0.0132 (5)	0.8624 (2)	0.0510 (9)	
C3	0.9574 (2)	0.1578 (5)	0.8973 (2)	0.0485 (9)	
C4	1.0540 (3)	0.2126 (6)	0.9250 (2)	0.0674 (11)	
H4A	1.0702	0.3263	0.9494	0.081*	
C5	1.1250 (3)	0.0870 (7)	0.9140 (3)	0.0735 (12)	
H5A	1.1903	0.1195	0.9309	0.088*	
C6	1.1036 (3)	−0.0738 (6)	0.8811 (3)	0.0687 (11)	
H6A	1.1534	−0.1538	0.8760	0.082*	
C7	1.0072 (3)	−0.1268 (5)	0.8534 (2)	0.0538 (9)	
C8	0.9868 (3)	−0.3022 (6)	0.8160 (3)	0.0743 (13)	
C9	−0.0871 (3)	0.2856 (5)	0.4071 (3)	0.0707 (11)	
H9A	−0.0390	0.3709	0.4351	0.106*	
H9B	−0.1311	0.2595	0.4455	0.106*	
H9C	−0.1228	0.3360	0.3531	0.106*	
C10	−0.0378 (3)	0.1138 (5)	0.3880 (2)	0.0472 (8)	
C11	−0.0849 (3)	−0.0391 (6)	0.3503 (2)	0.0573 (10)	
C12	−0.0389 (3)	−0.1889 (6)	0.3260 (2)	0.0669 (11)	
H12A	−0.0752	−0.2850	0.2977	0.080*	
C13	0.0618 (3)	−0.1963 (6)	0.3435 (3)	0.0733 (12)	
H13A	0.0943	−0.2984	0.3295	0.088*	
C14	0.1137 (3)	−0.0442 (5)	0.3834 (2)	0.0609 (10)	
H14A	0.1814	−0.0446	0.3950	0.073*	
C15	0.0650 (3)	0.1033 (5)	0.4049 (2)	0.0509 (9)	
C16	0.1190 (3)	0.2561 (6)	0.4443 (3)	0.0646 (11)	

### Atomic displacement parameters (Å^2^)

**Table:**

	*U*^11^	*U*^22^	*U*^33^	*U*^12^	*U*^13^	*U*^23^
O1	0.115 (3)	0.095 (3)	0.102 (3)	0.006 (2)	0.033 (2)	−0.014 (2)
O2	0.098 (3)	0.108 (3)	0.108 (3)	0.018 (2)	0.016 (2)	−0.001 (2)
O3	0.099 (3)	0.144 (4)	0.106 (3)	−0.008 (3)	0.018 (2)	−0.010 (3)
O4	0.076 (2)	0.106 (3)	0.102 (2)	−0.0009 (19)	0.0258 (18)	−0.004 (2)
N1	0.115 (3)	0.071 (3)	0.087 (3)	0.004 (2)	0.020 (2)	−0.012 (2)
N2	0.079 (2)	0.064 (2)	0.065 (2)	0.014 (2)	0.0264 (19)	0.003 (2)
N3	0.089 (3)	0.085 (3)	0.101 (3)	−0.008 (2)	0.023 (2)	−0.017 (2)
N4	0.075 (3)	0.097 (3)	0.084 (3)	−0.011 (2)	0.020 (2)	0.005 (3)
C1	0.064 (3)	0.116 (4)	0.106 (4)	−0.020 (3)	0.037 (3)	−0.017 (3)
C2	0.056 (2)	0.053 (2)	0.052 (2)	−0.0032 (17)	0.0298 (17)	0.0022 (17)
C3	0.055 (2)	0.050 (2)	0.0460 (19)	0.0118 (17)	0.0226 (15)	−0.0005 (17)
C4	0.062 (2)	0.071 (3)	0.073 (3)	−0.007 (2)	0.0210 (19)	0.002 (2)
C5	0.051 (2)	0.091 (3)	0.083 (3)	−0.006 (2)	0.021 (2)	0.003 (3)
C6	0.068 (3)	0.076 (3)	0.074 (3)	0.016 (2)	0.041 (2)	0.008 (2)
C7	0.072 (2)	0.045 (2)	0.054 (2)	0.0078 (18)	0.0355 (18)	0.0067 (17)
C8	0.099 (4)	0.056 (3)	0.072 (3)	0.006 (3)	0.027 (2)	0.005 (2)
C9	0.070 (3)	0.067 (3)	0.081 (3)	0.009 (2)	0.031 (2)	−0.004 (2)
C10	0.059 (2)	0.0440 (19)	0.0454 (18)	0.0023 (17)	0.0260 (16)	0.0087 (16)
C11	0.053 (2)	0.069 (2)	0.053 (2)	−0.0123 (19)	0.0170 (16)	0.0077 (19)
C12	0.084 (3)	0.056 (2)	0.066 (2)	−0.021 (2)	0.029 (2)	−0.006 (2)
C13	0.091 (3)	0.064 (3)	0.075 (3)	0.000 (2)	0.041 (2)	−0.008 (2)
C14	0.058 (2)	0.065 (2)	0.064 (2)	0.0003 (19)	0.0224 (18)	−0.007 (2)
C15	0.055 (2)	0.054 (2)	0.048 (2)	−0.0040 (18)	0.0199 (16)	0.0038 (17)
C16	0.064 (3)	0.060 (3)	0.078 (3)	−0.004 (2)	0.034 (2)	−0.014 (2)

### Geometric parameters (Å, °)

**Table:**

O1—N2	1.169 (4)	C5—H5A	0.9300
O2—N2	1.224 (4)	C6—C7	1.388 (5)
O3—N4	1.182 (5)	C6—H6A	0.9300
O4—N4	1.200 (5)	C7—C8	1.425 (6)
N1—C8	1.063 (5)	C9—C10	1.504 (5)
N2—C3	1.483 (4)	C9—H9A	0.9600
N3—C16	1.125 (5)	C9—H9B	0.9600
N4—C11	1.518 (5)	C9—H9C	0.9600
C1—C2	1.485 (5)	C10—C11	1.376 (5)
C1—H1A	0.9600	C10—C15	1.414 (5)
C1—H1B	0.9600	C11—C12	1.372 (5)
C1—H1C	0.9600	C12—C13	1.384 (5)
C2—C3	1.399 (5)	C12—H12A	0.9300
C2—C7	1.410 (5)	C13—C14	1.410 (5)
C3—C4	1.396 (5)	C13—H13A	0.9300
C4—C5	1.396 (6)	C14—C15	1.363 (5)
C4—H4A	0.9300	C14—H14A	0.9300
C5—C6	1.304 (6)	C15—C16	1.426 (5)
			
O1—N2—O2	120.7 (4)	C6—C7—C8	119.0 (4)
O1—N2—C3	121.9 (4)	C2—C7—C8	119.0 (4)
O2—N2—C3	117.4 (4)	N1—C8—C7	171.6 (6)
O3—N4—O4	122.9 (5)	C10—C9—H9A	109.5
O3—N4—C11	116.7 (4)	C10—C9—H9B	109.5
O4—N4—C11	119.0 (4)	H9A—C9—H9B	109.5
C2—C1—H1A	109.5	C10—C9—H9C	109.5
C2—C1—H1B	109.5	H9A—C9—H9C	109.5
H1A—C1—H1B	109.5	H9B—C9—H9C	109.5
C2—C1—H1C	109.5	C11—C10—C15	114.6 (3)
H1A—C1—H1C	109.5	C11—C10—C9	125.2 (3)
H1B—C1—H1C	109.5	C15—C10—C9	120.2 (3)
C3—C2—C7	114.3 (3)	C12—C11—C10	124.5 (4)
C3—C2—C1	125.0 (4)	C12—C11—N4	117.8 (4)
C7—C2—C1	120.7 (4)	C10—C11—N4	117.7 (4)
C4—C3—C2	124.2 (3)	C11—C12—C13	119.7 (4)
C4—C3—N2	116.7 (3)	C11—C12—H12A	120.1
C2—C3—N2	119.1 (3)	C13—C12—H12A	120.1
C3—C4—C5	116.3 (4)	C12—C13—C14	118.0 (4)
C3—C4—H4A	121.9	C12—C13—H13A	121.0
C5—C4—H4A	121.9	C14—C13—H13A	121.0
C6—C5—C4	122.7 (4)	C15—C14—C13	120.3 (4)
C6—C5—H5A	118.7	C15—C14—H14A	119.9
C4—C5—H5A	118.7	C13—C14—H14A	119.9
C5—C6—C7	120.7 (4)	C14—C15—C10	122.8 (3)
C5—C6—H6A	119.7	C14—C15—C16	119.3 (3)
C7—C6—H6A	119.7	C10—C15—C16	117.9 (3)
C6—C7—C2	121.9 (4)	N3—C16—C15	174.6 (5)

### Hydrogen-bond geometry (Å, °)

**Table:**

*D*—H···*A*	*D*—H	H···*A*	*D*···*A*	*D*—H···*A*
C1—H1A···O2	0.96	2.08	2.768 (6)	128
C1—H1C···O2^i^	0.96	2.38	3.229 (6)	147
C4—H4A···O1^ii^	0.93	2.47	3.390 (6)	171
C9—H9B···O4	0.96	2.35	2.831 (5)	110
C9—H9C···O3^iii^	0.96	2.50	3.400 (4)	156

Symmetry codes: (i) −*x*+3/2, *y*−1/2, −*z*+3/2; (ii) −*x*+2, −*y*+1, −*z*+2; (iii) −*x*−1/2, *y*+1/2, −*z*+1/2.
